# Enhancing robot evolution through Lamarckian principles

**DOI:** 10.1038/s41598-023-48338-4

**Published:** 2023-11-30

**Authors:** Jie Luo, Karine Miras, Jakub Tomczak, Agoston E. Eiben

**Affiliations:** 1 Vrije Universiteit Amsterdam, Amsterdam, The Netherlands; 2 Eindhoven University of Technology, Eindhoven, The Netherlands

**Keywords:** Computational science, Computer science

## Abstract

Evolutionary robot systems offer two principal advantages: an advanced way of developing robots through evolutionary optimization and a special research platform to conduct what-if experiments regarding questions about evolution. Our study sits at the intersection of these. We investigate the question “What if the 18th-century biologist Lamarck was not completely wrong and individual traits learned during a lifetime could be passed on to offspring through inheritance?” We research this issue through simulations with an evolutionary robot framework where morphologies (bodies) and controllers (brains) of robots are evolvable and robots also can improve their controllers through learning during their lifetime. Within this framework, we compare a Lamarckian system, where learned bits of the brain are inheritable, with a Darwinian system, where they are not. Analyzing simulations based on these systems, we obtain new insights about Lamarckian evolution dynamics and the interaction between evolution and learning. Specifically, we show that Lamarckism amplifies the emergence of ‘morphological intelligence’, the ability of a given robot body to acquire a good brain by learning, and identify the source of this success: newborn robots have a higher fitness because their inherited brains match their bodies better than those in a Darwinian system.

## Introduction

Evolutionary robotics (ER) is a research field that applies Evolutionary algorithms (EAs) to design and optimize the body, the brain, or both, for simulated and real autonomous robots^1,2^. It is a promising area with a powerful rationale: as natural evolution has produced successful life forms for practically all possible environmental niches on Earth, it is plausible that artificial evolution can produce specialized robots for various environments and tasks.

Early studies in ER were one-sided in the sense that they explored the evolution of the controller (brain) only, while the morphologies (bodies) were fixed^3,4^. A holistic approach—the conjoint evolution of morphology and controller—was introduced by Karl Sims in his seminal work with virtual creatures^5^ that inspired several studies since then^6–15^. Extending the scope of evolution to include the bodies as well as a fundamental step towards complex robotic intelligence, but there is (at least) one more layer that should be considered: learning^16^. Learning allows to fine-tune the coupling between body and brain and it provides extra means for adapting to the environment. Here again, the majority of related work consists of applying learning algorithms to the evolvable brains of robots with fixed bodies^17–26^. However, it has been argued that morphological robot evolution must include a learning stage immediately after reproduction^27^, and some recent studies have investigated the combination of body evolution, brain evolution, and learning^28–36^.

Further to being a bio-inspired technique for developing robots, ER forms an alternative approach to studying issues in evolutionary biology. An evolving robot system, be it real or simulated, can be considered as a model of an evolving system of living organisms and used to test hypotheses experimentally^37^. ER has been used in this manner to investigate, for example, the evolution of cooperation, whether altruistic^38,39^ or not^40^, the evolution of communication^41–43^, morphological complexity^44,45^, and collective swarming^46^. Such studies can be positioned in the broader category of biorobotics, where robots are employed in experiments for the study of animal and human behaviour^47–49^.

Strictly speaking, our current work does not fall in this category since we are not modelling any existing life form. Instead, we follow John Maynard Smith, one of the fathers of modern theoretical biology, who argued: “So far, we have been able to study only one evolving system, and we cannot wait for interstellar flight to provide us with a second. If we want to discover generalizations about evolving systems, we will have to look at artificial ones.”^50^. Research along this line of thought has a what-if character; it is not about Life as we know it but Life as it could be. Specifically, in this paper, we do not try to emulate an existing biological phenomenon but implement and investigate one that is possible in robots, while it may not exist in living organisms: Lamarckism.

Lamarckism is one of the most enduring controversial matters in evolutionary biology asserting that adaptations acquired by an individual during its lifetime can be inherited by its offspring^51^. Although this theory has been disproven by modern genetics, the concept of Lamarckian evolution is still a subject of debate and there is no clear consensus on whether it may occur in some form in nature^52^. For instance, some argue that epigenetic changes^53^ represent a form of Lamarckism.

By simulating Lamarckian evolution in artificial creatures we can conduct a thought experiment: What if Lamarck was not completely wrong and individual traits acquired during a lifetime could be inherited by the offspring? Empirical data delivered by computer simulations allows for analyzing the evolutionary dynamics and exploring the potential benefits and drawbacks of Lamarckian evolution for developing robots. From the robotics perspective, this contributes to designing more advanced evolutionary algorithms that in turn can deliver better robotic systems. From the biological perspective, this delivers new insights into possible evolutionary dynamics, demonstrated in a system that is artificial, but evolutionary since it features selection and reproduction with heredity.

Previous research on artificial evolution combined with learning is mostly limited to Darwinian systems^15,33,36,54^ and the Baldwin effect^29,30^. The few existing studies on Lamarckian evolution can be divided into three categories. First, disembodied evolution applying an evolutionary algorithm to machine learning techniques^55–63^. In this category, studies found that the Lamarckian mechanism quickly yields good solutions, accelerating convergence and adapting to dynamic environments but may risk converging to a local optimum. Second, embodied evolution of controllers for robots with fixed bodies^23,64,65^. In this category, studies found that Lamarckian evolution is effective in improving the performance of robot controller evolution, and that the learning process can reduce the negative impact of the simulation-reality gap. Finally, there is the full-blown case, embodied evolution of morphologies and controllers together in a Lamarckian manner. This is the most complex category that has hardly been studied so far with only two papers we are aware of^66,67^. These considered the simplest possible robot task (undirected locomotion or gait learning) and observed the increased efficiency and efficacy of Lamrackian evolution without analyzing the underlying mechanisms and the source of the improved performance.

In summary, previous studies focused on establishing the advantages of Lamarckism without a deeper investigation into why and how Lamarckism delivers such benefits and to date there is hardly any knowledge about the most complex case of morphologically evolvable robots. This latter may be rooted in the difficulty in designing and implementing such a system. Technically speaking, it requires a reversible mapping between (certain segments of) the genotype and the phenotype. In particular, some features of the robot controller must be evolvable (i.e. inheritable) as well as learnable, and the traits acquired by the learning algorithm during the lifetime of a robot must be coded back to the genotype to make them inheritable.

In this work, we investigate the effects of Lamarckism on morphologically evolvable robots. Specifically, we solve the reversible genotype-phenotype mapping problem and create a Lamarckian system, where certain phenotypic controller features learned during lifetime (after birth) can be coded back to the robots’ genotypes, thus making them inheritable by the offspring. We compare the Lamarckian system to a Darwinian system^36^ in which learning occurs in the same way, but learned traits are not inheritable. All mechanisms and parameters are the same in both systems, with the exception of the inheritance of learned traits.

The main contributions of the present work are twofold: (a) a general framework for a Lamarckian robot evolution system with a reversible genotype-phenotype mapping, and (b) novel insights into the deeper effects of Lamarckism underlying its increased effectiveness and efficiency.

## Results

The robot evolution system behind the results is based on modular robots composed of passive blocks and active hinges forming the body and a network of Central Pattern Generators (CPGs) as a brain, see the Methods section for details. Bodies and brains both undergo evolution. Thus, the robotic phenotypes consist of a body, being a specific configuration of modules, and a brain, which is a CPG network where each hinge has one corresponding CPG that drives it and CPGs of neighbouring modules are connected by design. Note that the topology of the brain is fully determined by the body, leaving the connection weights the only evolvable component of the brain. Hence, the genotypes consist of a body-coding segment that specifies the modules’ configuration and a brain-coding segment that defines the weights of the CPG network. As we are using the generic robot evolution architecture proposed in^27^, all newborn robots undergo a learning process that aims to optimize the inherited weights. The fitness of a robot is evaluated after the learning process, hence selection is based on the behaviour (task performance) obtained with the learned weights. In the Darwinian version of our system, the learned weights are part of the phenotypes, but do not affect the genotypes. This means that the original, inherited weights of the robot parents are used when producing the weights for the brain of the robot offspring. In contrast, in the Lamarckian version of our system, the brain genotypes are changed by learning: the learned weights overwrite the inherited ones in the genotype of the learner. Thus, the weights for the brain of the robot offspring are produced by using the learned weights of the robot parents.

### Task performance

Robots are evolved for a point navigation task, requiring that the robot visits a sequence of target points (see the Methods section for details). Their task ability is used as the fitness function for evolution and as the reward function for the lifetime learning method, cf. Algorithm 1.

**Figure 1:**
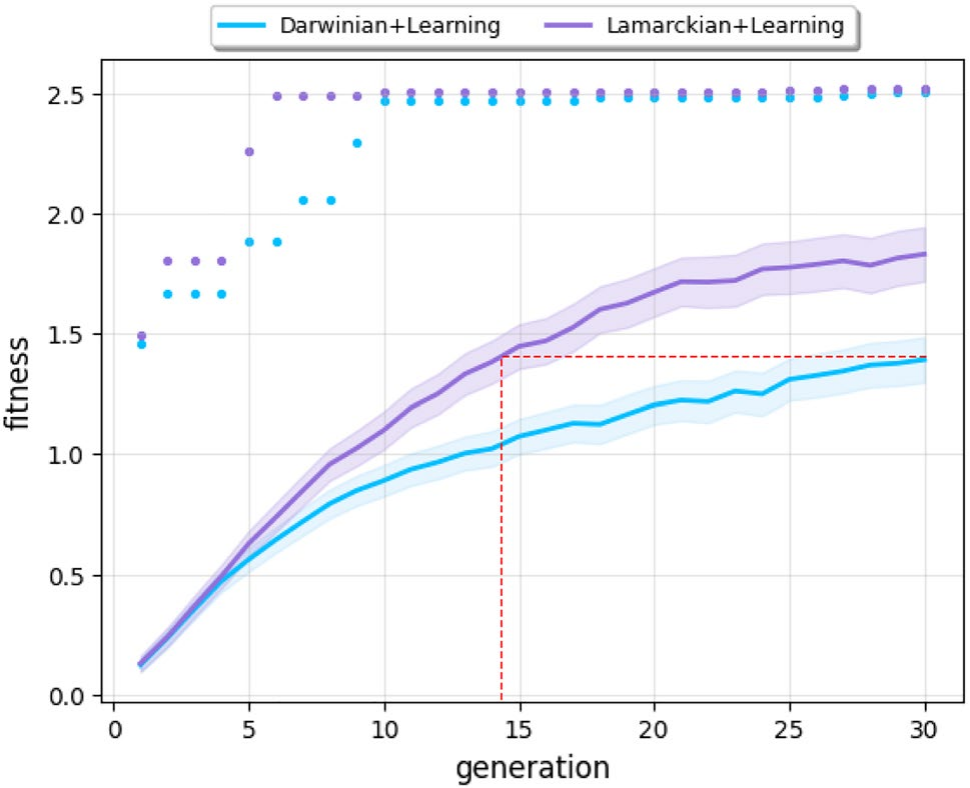
Mean (lines) and maximum (dots) fitness over 30 generations. The bands indicate the 95% confidence intervals (Sample Mean ± t-value $\times$ Standard Error).

Figure 1 exhibits the development of fitness over consecutive generations of the Lamarckian and the Darwinian systems. These curves show that the best robots that the Darwinian system produces reach a fitness of 2.5, but the populations produced by the Lamarckian system are significantly better—approximately 25% higher at the end.

**Figure 2:**
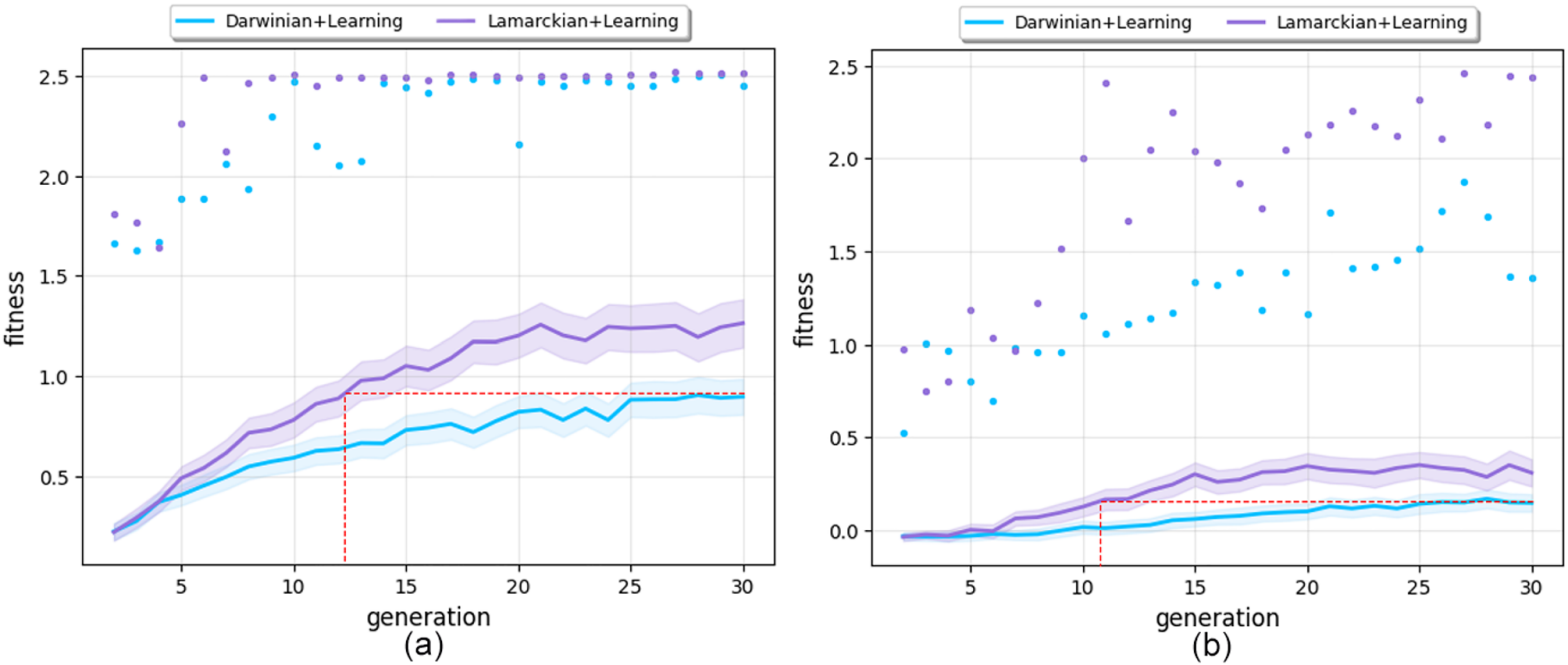
Mean (lines) and maximum (dots) fitness of newborn robots after learning (**a**) and before learning (**b**) over 30 generations. The bands indicate the 95% confidence intervals (Sample Mean ± t-value $\times$ Standard Error). Mean (lines) and maximum (dots) fitness of newborn robots after learning (**a**) and before learning (**b**) over 30 generations. The bands indicate the 95% confidence intervals.

**Figure 3:**
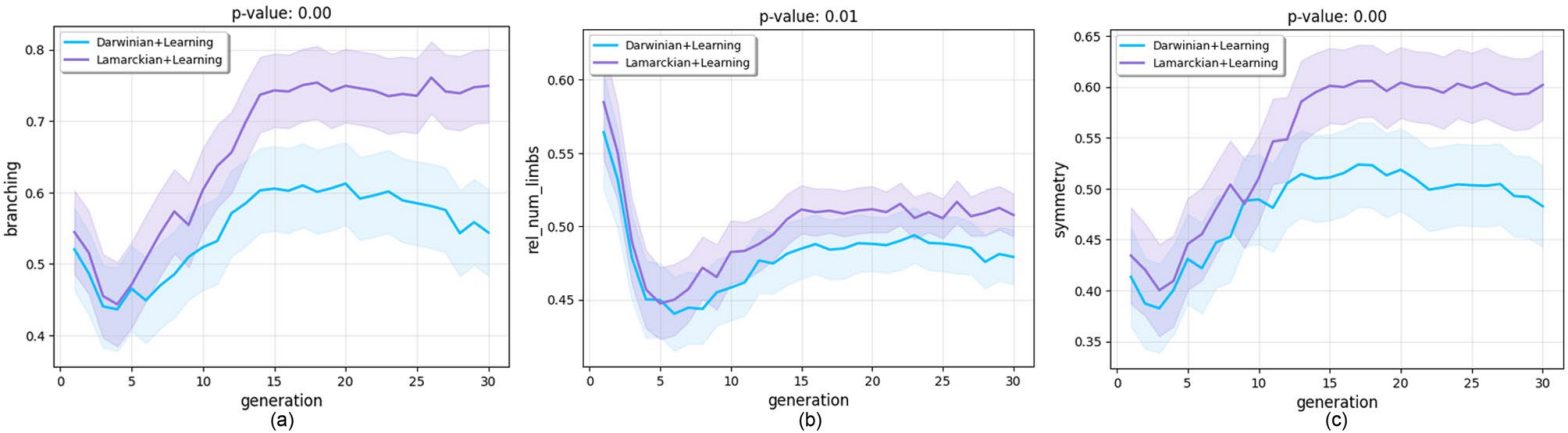
Morphological traits over generations. We present the progression of their means averaged over 20 runs for the entire population. Shaded regions denote a 95% confidence interval. The *p*-values in the title correspond to the final generations. The significance level after Bonferroni correction for 6 comparisons is p < 0.006.

**Figure 4:**
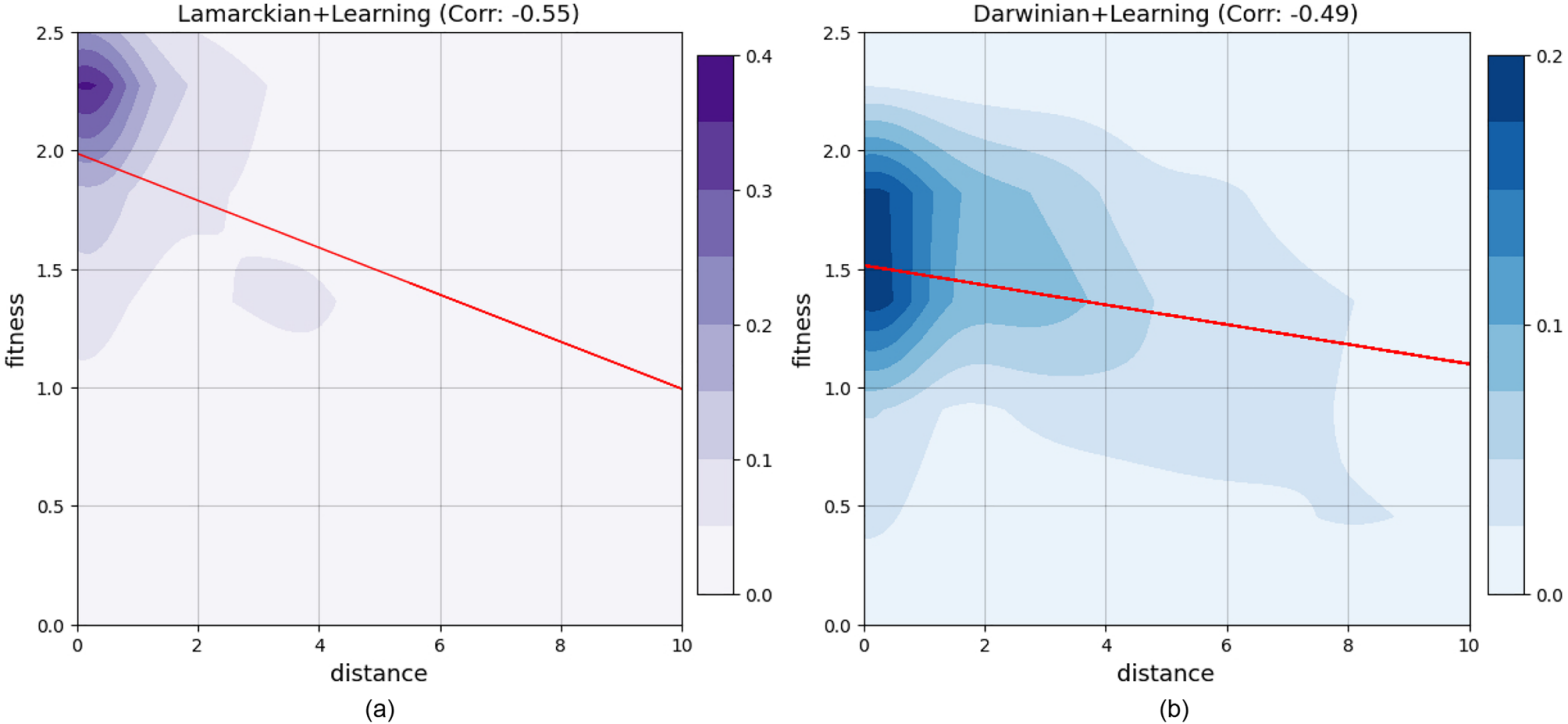
Density plots showing the relation between fitness and the morphological tree-edit distance between child and parent for the Lamarckian system (**a**) and the Darwinian system (**b**). The darker the colour, the higher density of the robots in that region. The red lines are the regression lines. The correlation efficiency rate is shown in the title of each plot.

**Figure 5:**
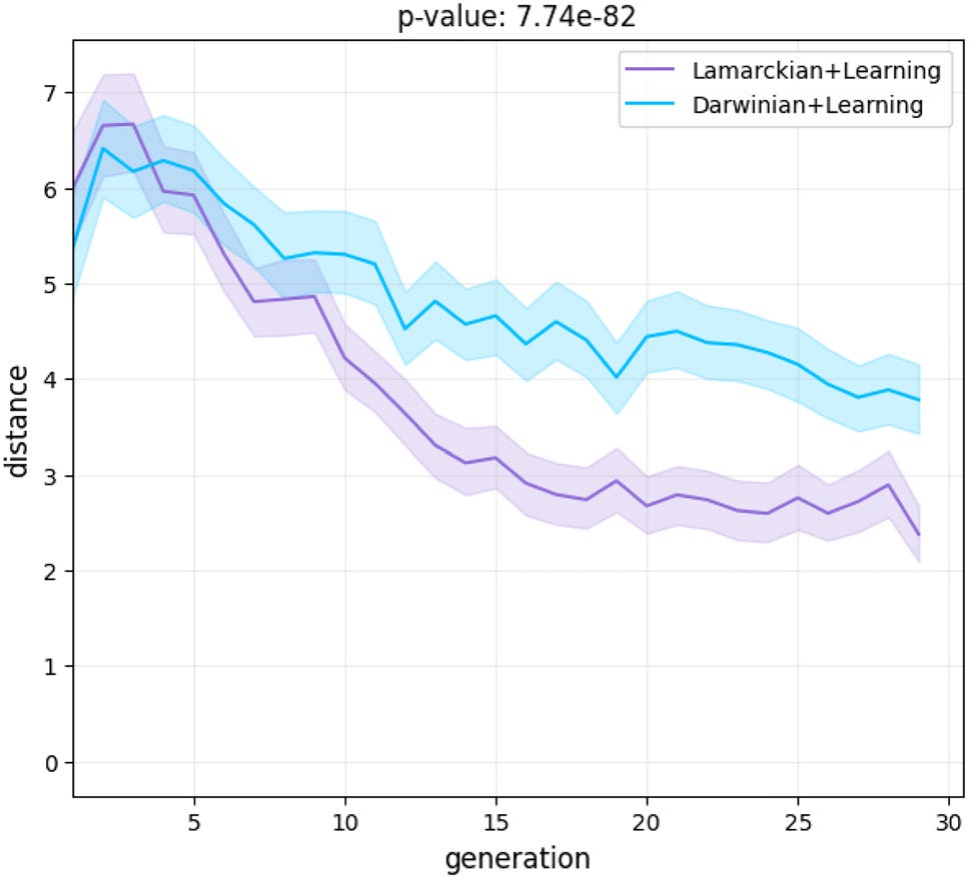
Morphological tree-edit distance over generations. Shaded regions denote the 95% confidence interval.

Figure 1 also demonstrates the differences in efficiency. The Lamarckian system is more efficient than the Darwinian one, as it finds the best solutions (robots with the highest fitness) much faster. Furthermore, the dotted red lines show that halfway through the run (around generation 14) the Lamarckian system has reached the quality produced by the Darwinian system only by the end of the evolutionary process. This can be seen as significant ‘savings’ of 2,240,000 evaluations (25 offspring $\cdot$ 16 generations $\cdot$ 280 learning trials $\cdot$ 20 runs).

To investigate more closely what allows the Lamarckian system to be more effective and efficient than the Darwinian, we inspected the fitness of the newborn robots both after learning (Fig. 2a) and before learning (Fig. 2b): not only are the Lamarckian parents better than the Darwinian parents after learning, but also before learning. These observations mean that not only are Lamarckian robots better after they learn, but also better immediately after they are born, suggesting that the performance superiority of the Lamarckian system derives from having had a better starting point.

Compared to the average fitness in Fig. 1, it is shown that the average fitness of the newborn robots is consistently lower. This discrepancy can be attributed to the following rationale: within the population of 50 individuals, comprising 25 survivors from the previous generation and 25 newborns (see the Evolution Process section), the survivors exhibit significantly higher fitness levels when compared to the newborns of the current generation. Consequently, the overall average fitness of the population exceeds that of the newborns and exhibits a steady increase with each subsequent generation.

### Robot morphologies

We analyze the morphological properties of the robots addressing four different aspects: the morphological traits (details about the measures can be found in^68^), the morphological similarity between offspring and parents, the morphological diversity at each generation, and the morphological intelligence.

#### Morphological traits

To investigate the morphologies generated by the Lamarckian and Darwinian evolution systems, we consider eight morphological traits to quantitatively analyze the evolved morphologies of all robots. Among these eight traits, only three of them presented significant differences (Fig. 3), namely branching, number of limbs, and symmetry. Robots evolved by the Lamarckian system tend to be more symmetric and have more branches and limbs than robots evolved with the Darwinian system. Nevertheless, despite these observed differences, visual inspection of the top bodies hardly allows for an intuitive differentiation between their shapes (Fig. 9). Moreover, a PCA analysis using these same eight traits does not show any difference between the morphologies produced by each method. Therefore, although there is evidence for differences in morphological traits, these differences appear marginal (Fig. 8).

#### Morphological parent-child similarity

We calculate the morphological similarity between a child and its fittest parent by the tree-edit distance of their morphological structures. To this end, we use the APTED algorithm, the state-of-the-art solution for computing the tree-edit distance^69^.

Figure 4 shows the correlation between fitness and distance. For both methods, the correlation between fitness and parent-child distance is negative. This means that the more similar the offspring is to the parent, the higher the fitness of the offspring. Importantly, this correlation is stronger in the case of the Lamarckian system.

Furthermore, Fig. 5 shows how the average distance progresses over the generations. With both systems, we see pressure for reducing the distance between the offspring and parent, but this pressure is higher with the Lamarckian system. This effect is logical because it is expected that the brain of a parent would be a better match to a body similar to its own body.

#### Morphological diversity

Morphological diversity is the morphological variety of each population using tree-edit distance. It is calculated as the average distance of the difference between any two robots d(x,y) at each generation.

**Figure 6:**
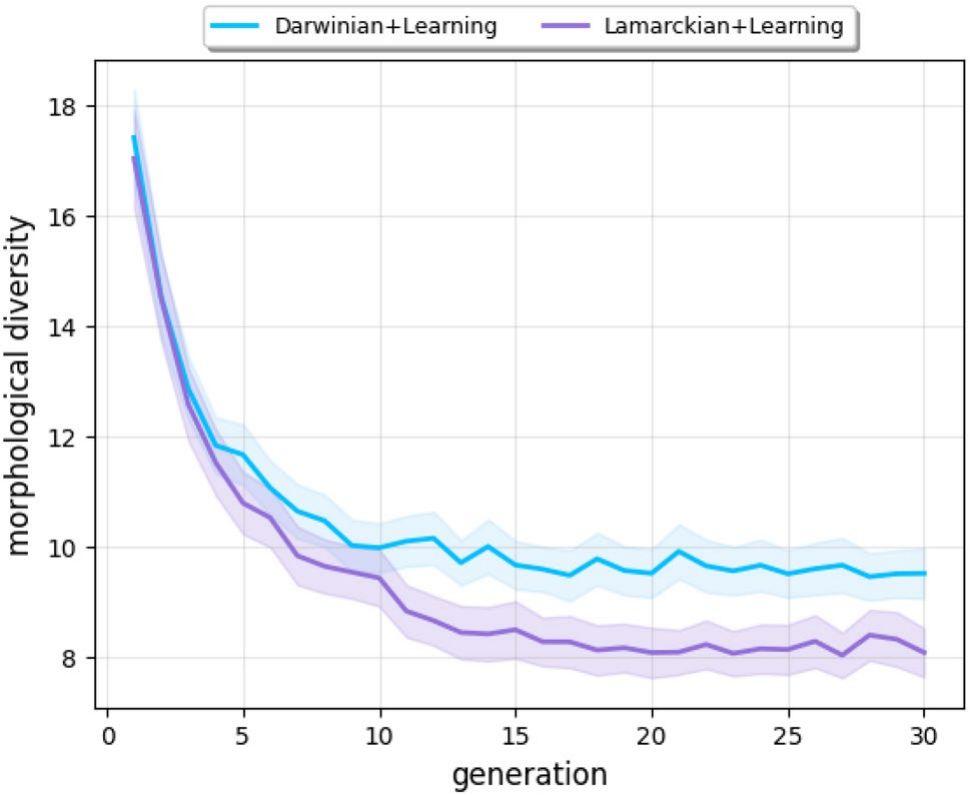
Progression of morphological diversity averaged over 20 runs. The bands indicate the 95% confidence intervals.

Figure 6 illustrates a notable trend: the morphological diversity of the Lamarckian system declines at a more rapid rate and reaches a lower level compared to the Darwinian system (*p*-value $6.5e-110$). This observation suggests that the Lamarckian system converges into superior bodies faster.

Considering that our current experimental setup uses a static environment, this earlier discovery of better body traits was beneficial: the Lamarckian system reached a much better result than the Darwinian system, almost achieving the global optimum. However, such a quick loss of diversity might not be beneficial if we consider a scenario where the environment is dynamic because it could lead the system to run out of genetic variation to cope with future changes.

#### Morphological intelligence

Morphology influences how the brain learns^70^. In our system, a robot body can be more suitable for learning good control than other morphologies, for instance, because its shape might reduce the computations that the controller needs to perform, or it could tolerate imperfect controller settings better. We conceptualize the potential of a morphology to facilitate the brain to learn a good controller as *morphological intelligence* and quantify it by the learning delta: the task performance after learning minus the task performance before learning.

**Figure 7:**
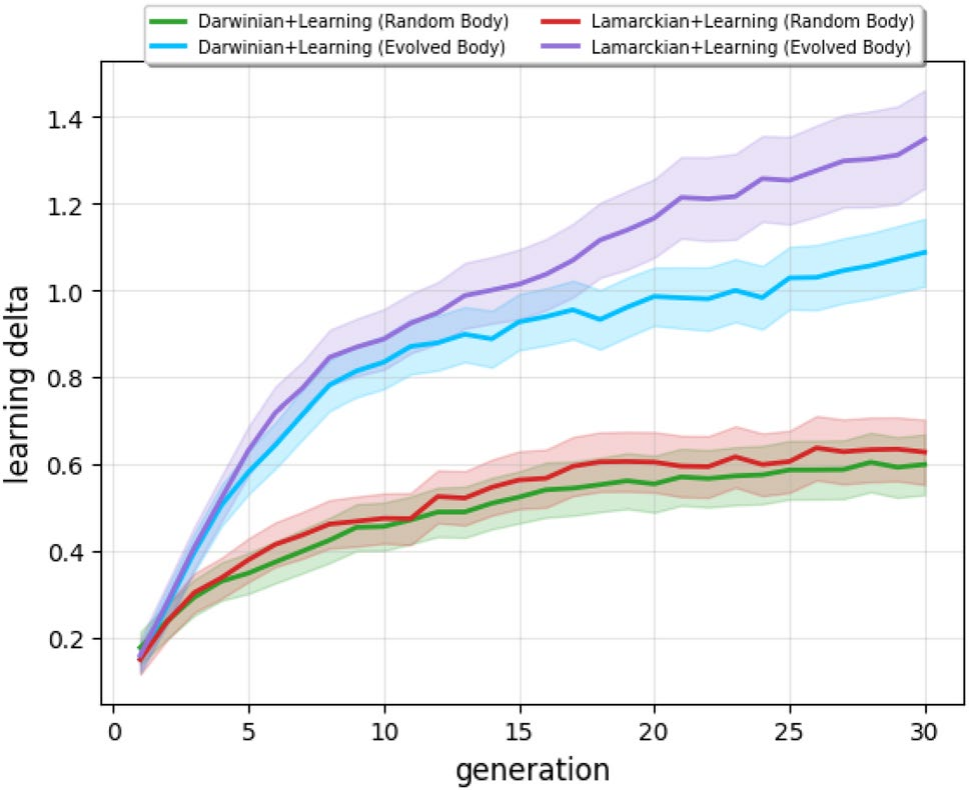
Progression of the learning delta using the two body setups averaged over 20 runs. The bands indicate the 95% confidence intervals.

To investigate morphological intelligence in our system, we conducted a control experiment using robots with randomly changing bodies between generations rather than evolving ones. Specifically, after performing reproduction, we replaced the inherited body with a randomly generated one while keeping the inherited brain in the offspring. Subsequently, we applied the learning procedure as before, improving the brain in the given random body, and coded back the learned weights into the brain genome for the Lamarckian variant. Figure 7 compares the main experiment with the control experiment and allows for three main observations. First, the learning delta is growing in both the evolvable and random body experiments. Second, the learning delta of random bodies is growing less steeply than that of the evolvable bodies. Third, the learning delta of the Lamarckian system in the evolvable body experiment grows more steeply than that of the Darwinian system.

Our conclusion is that the growth of the learning delta in the evolvable body experiments is caused by two effects. One effect is the increase of morphological intelligence—this is evidenced by the advantage of the truly evolutionary runs over the runs with random bodies. The other effect is manifested by the growth of the learning deltas despite using random bodies, and we attribute this to increasing controller robustness. When the learning delta grows from one generation to another, it means that the current population has a greater learning ability than the previous one. The fact that this happens despite having replaced the bodies of the offspring with random bodies shows that the evolved brain of an individual is robust to some extent, i.e., it can induce good task performance in an arbitrary body—one that it was not optimized for. Because of this robustness, the brains form a better starting point for learning. This better start affords the learning process to fine-tune the controller better and, therefore, reach a higher task performance—ultimately, a higher learning delta.

In summary, the control experiments indicate that the growth of the learning delta is not fully explained by the notion of morphological intelligence. We hypothesize that the other force at play is the increasing robustness of the evolved controllers, but a thorough analysis would require further research beyond the scope of this paper. Finally, we can observe that the Lamarckian system produces better newborn robots (Fig. 2-b) and a higher learning delta in the evolvable body experiments (in Fig. 7). These observations support the conclusion that Lamarckian evolution delivers better learning robots than its Darwinian counterpart.

**Figure 8:**
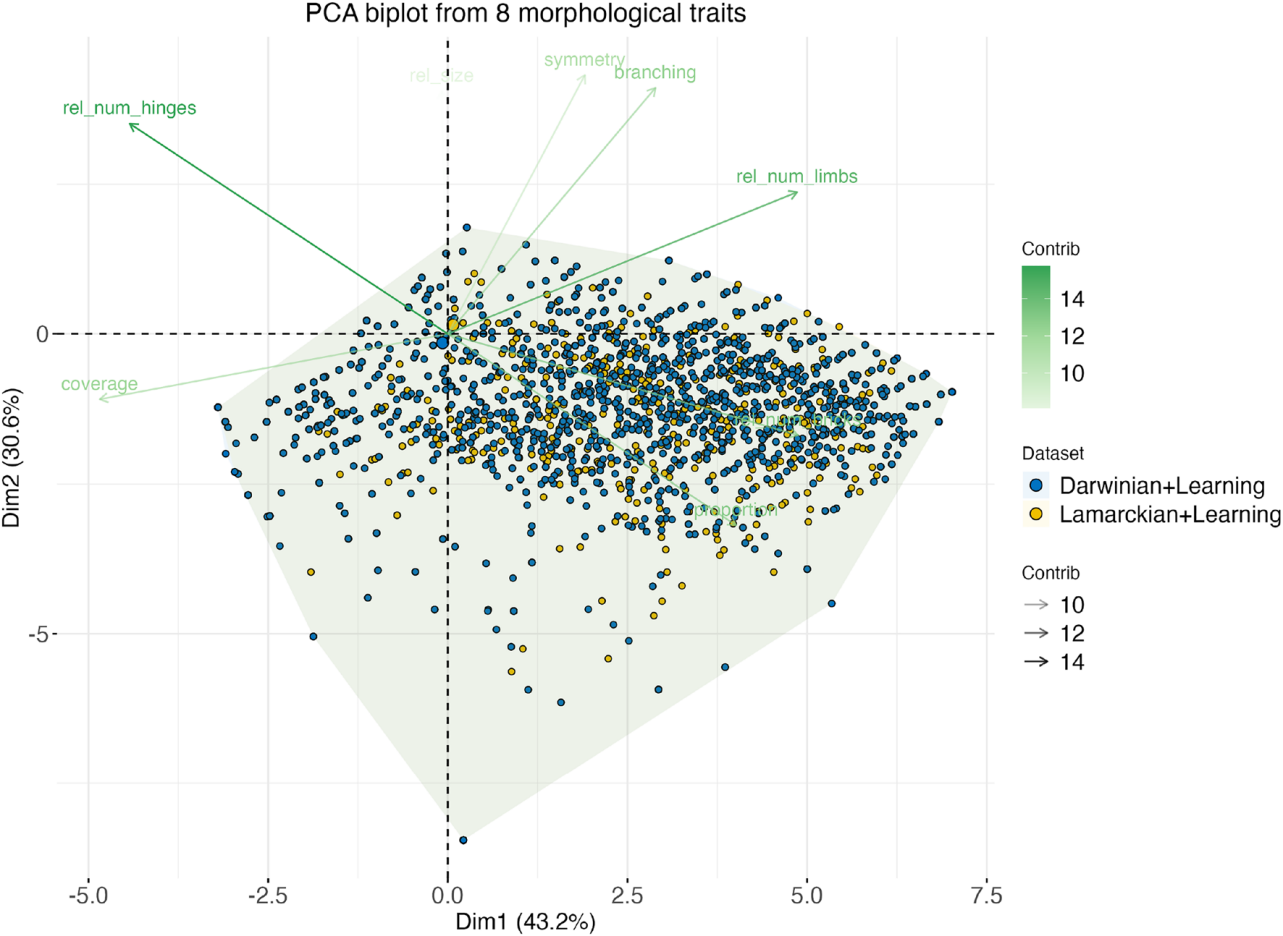
Principal Component Analysis (PCA) biplot showing the distribution of samples based on 8 morphological traits in datasets of both methods. Each point represents a robot sample, and the plot displays the first two principal components (Dim1 and Dim2), which explain 43.2% and 30.6% of the total variance, respectively. Furthermore, the biplot displays the variables (morphological traits) as arrows, representing their contribution to the principal components. Traits pointing in similar directions are co-regulated or have similar expression patterns across the samples.

**Figure 9:**
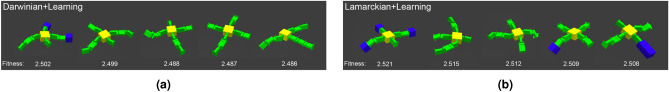
The 5 best robots produced by both methods.

### Robot behavior

**Figure 10:**
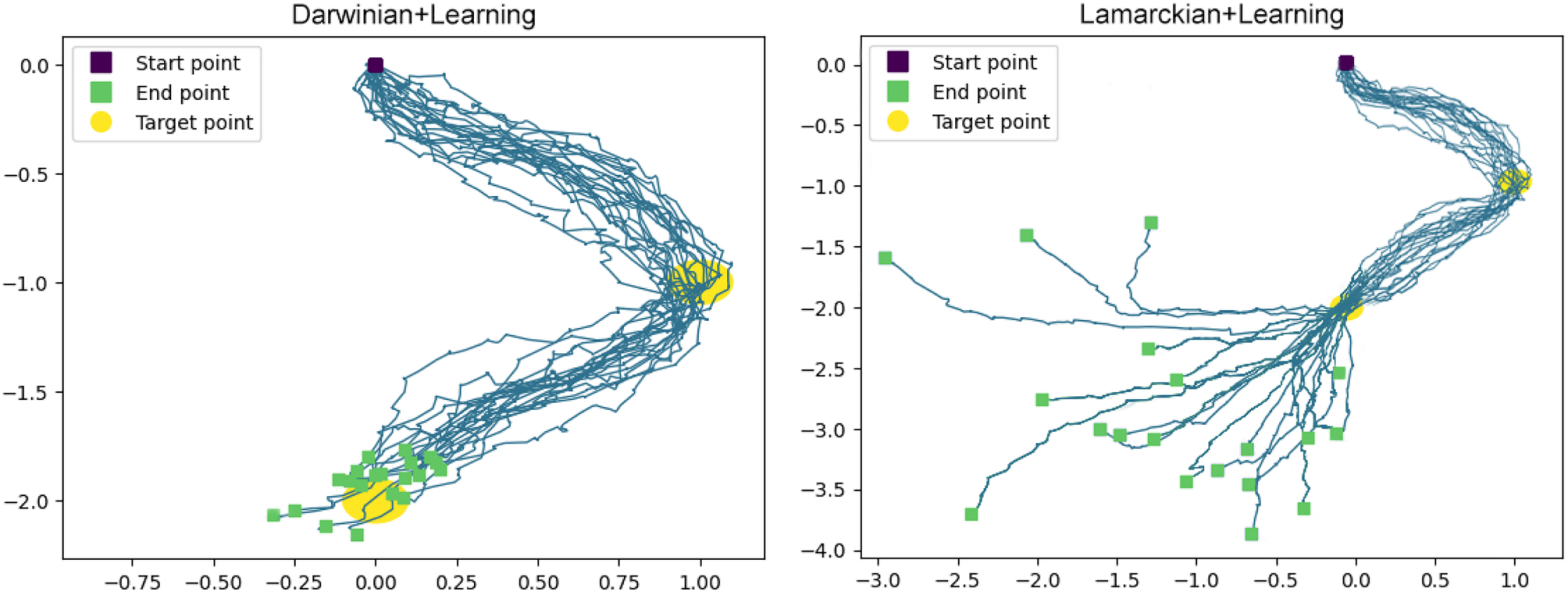
Trajectories of the best 20 robots from both methods in the point navigation task. The purple square is the starting point. Two yellow circles are the target points which robots aim to go through. The blue lines are the trajectories of robots ending at the green squares.

To obtain a better understanding of the robots’ behaviour, we visualize the trajectories of the 20 best-performing robots from both methods in the last generation across all runs. Figure 10 shows that all robots from the Lamarckian system reached the two target points much earlier than the ones from the Darwinian system. This can be concluded because after reaching the target, they still have time to keep moving further from the target.

## Discussion

This investigation exceeds existing studies about Lamarckism in (simulated) robot evolution systems. It is not limited to evolving brains for fixed bodies, but considers Lamarckism in the most complex case, where morphologies and controllers both undergo evolution. A key feature of the system is the invertible genotype-phenotype mapping regarding the robot brains. This is an essential prerequisite for a Lamarckian system, because learning always acts on the phenotypes, after birth. If the genotype-phenotype mapping is invertible, then the newly learned traits that were not present in the robot at birth can be coded back to its genotype before it reproduces. This makes the learned traits inheritable, thus evolvable. Our system is based on modular robots whose body configuration is evolvable, together with their brains. While in our current system the number of different modules is limited, the principle behind our design is generic, and applicable for robots with more different modules. The only specific feature our solution exploits is that the controller architecture can be derived from the morphology. Hereby the search space of possible robot brains becomes parameterized and representable by a tensor.

The first set of our findings reconfirms earlier results about the increased efficiency and efficacy of Lamarckian evolution. Specifically, we showed that the Lamarckian system reaches the top fitness levels of the Darwinian system with just half of the effort (Fig. 1). Additionally, it presents a higher overall efficacy: the average fitness of the final populations is 25% higher when using the Lamarckian system. Previous work, from ourselves, with similar findings was based on much simpler robots (without sensors using open-loop controllers) and a simple task (undirected gait learning)^66,67^. Here, we move the front of applicability, showing that Lamarckian evolution is also superior in practically more relevant cases: robots with sensors and closed-loop controllers, evolved for a more challenging task.

A novel insight of this paper is that the newborn robots in the Lamarckian system are better even before the learning process is performed. Despite sounding logical, this observation is not trivial. Although simple logic would posit that offspring with inherited ‘knowledge’ will start learning from a higher region of the search space, this may not be true. For instance, it could be the case that the newborn robots are not particularly better and that the advantage of Lamarckism lies in the strategic initialization of the learning process. That is to say, Lamarckian offspring start in a region that is not necessarily higher, but better for a successful learning process. This study is the first to investigate this issue and provide empirical data about it.

Furthermore, we showed hitherto unknown differences between the evolution of morphologies in a Lamarckian and a Darwinian system. Although the fittest morphologies are similar to each other (Fig. 8, 9), the two systems differ in the morphologies created during evolution. In particular, in the Lamarckian system the body of the offspring is more similar to the parents (Fig. 4, 5) and the population converged to superior bodies faster (Fig. 6).

Another new insight about Lamarckian evolution was revealed by using the notion of the learning delta, the increase of performance achieved by learning after birth. This is a specific concept for morphological robot evolution combined with learning. In such systems, the fitness of a newborn robot can be measured right after birth with its inherited body and brain and compared with the fitness of the same body with the learned brain. Figure 7 indicates the emergence of morphological intelligence: over the course of evolution, the robots are becoming better learners in the Darwinian as well as the Lamarckian system. Our data also shows that this effect is greater in the case of Lamarckian evolution. However, our experiments raised new questions regarding this difference and even about the learning deltas. Specifically, Fig. 3 shows morphological convergence after about generation 15, whereas the learning delta is still growing (Fig. 7). This could be explained by some ‘hidden’ properties of the morphologies that are relevant for learning, but not captured by the morphological traits we are monitoring here. The observation that the morphological diversity levels reach a plateau but do not drop to zero (Fig. 6) corroborates this thought, but further research is needed to shed light on this issue.

To conclude, let us address some limitations of this work. The present study uses small population sizes due to the high computational costs involved. Furthermore, we only investigated a static environment. The effects of Lamarckism when environmental conditions change are completely unexplored to date. In principle, the effects can be positive (more rapid adaptation) or negative (over-fitting to conditions that no longer hold), making work about this potantially very interesting. Finally, we experimented only with simulated robots; applying the Lamarckian system to physical robots and testing their performance in diverse environments would provide valuable insights into the practical applications of our findings.

## Methods

### Robot morphology (body)

#### Body phenotype

The phenotype of the body is a subset of RoboGen’s 3D-printable components^71^: a morphology consists of one core component, one or more brick components, and one or more active hinges. The phenotype follows a tree structure, with the core module being the root node from which further components branch out. Child modules can be rotated 90 degrees when connected to their parent, making 3D morphologies possible. The resulting bodies are suitable for both simulation and physical robots through 3D printing.

#### Body genotype

The phenotype of bodies is encoded in a Compositional Pattern Producing Network (CPPN) which was introduced by Stanley^72^ and has been successfully applied to the evolution of both 2D and 3D robot morphologies in prior studies as it can create complex and regular patterns. The structure of the CPPN has four inputs and five outputs. The first three inputs are the x, y, and z coordinates of a component, and the fourth input is the distance from that component to the core component in the tree structure. The first three outputs are the probabilities of the modules being a brick, a joint, or empty space, and the last two outputs are the probabilities of the module being rotated 0 or 90 degrees. For both module type and rotation the output with the highest probability is always chosen; randomness is not involved.

The body’s genotype to phenotype mapping operates as follows: The core component is generated at the origin. We move outwards from the core component until there are no open sockets(breadth-first exploration), querying the CPPN network to determine the type and rotation of each module. Additionally, we stop when ten modules have been created. The coordinates of each module are integers; a module attached to the front of the core module will have coordinates (0,1,0). If a module would be placed on a location already occupied by a previous module, the module is simply not placed and the branch ends there. In the evolutionary loop for generating the body of offspring, we use the same mutation and crossover operators as in MultiNEAT (https://github.com/MultiNEAT/).

### Robot controller (brain)

#### Brain phenotype

We use a Central Pattern Generators (CPGs)-based controller to drive the modular robots, which has demonstrated their success in controlling various types of robots, from legged to wheeled ones in previous research. Each joint of the robot has an associated CPG that is defined by three neurons: an $x_i$-neuron, a $y_i$-neuron and an $out_i$-neuron. The change of the $x_i$ and $y_i$ neurons’ states with respect to time is obtained by multiplying the activation value of the opposite neuron with the corresponding weight $\dot{x}_i = w_i y_i$, $\dot{y}_i = -w_i x_i$. To reduce the search space we set $w_{x_iy_i}$ to be equal to $-w_{y_ix_i}$ and call their absolute value $w_i$. The resulting activations of neurons $x_i$ and $y_i$ are periodic and bounded. The initial states of all *x* and *y* neurons are set to $\frac{\sqrt{2}}{2}$ because this leads to a sine wave with amplitude 1, which matches the limited rotating angle of the joints.

**Figure 11:**
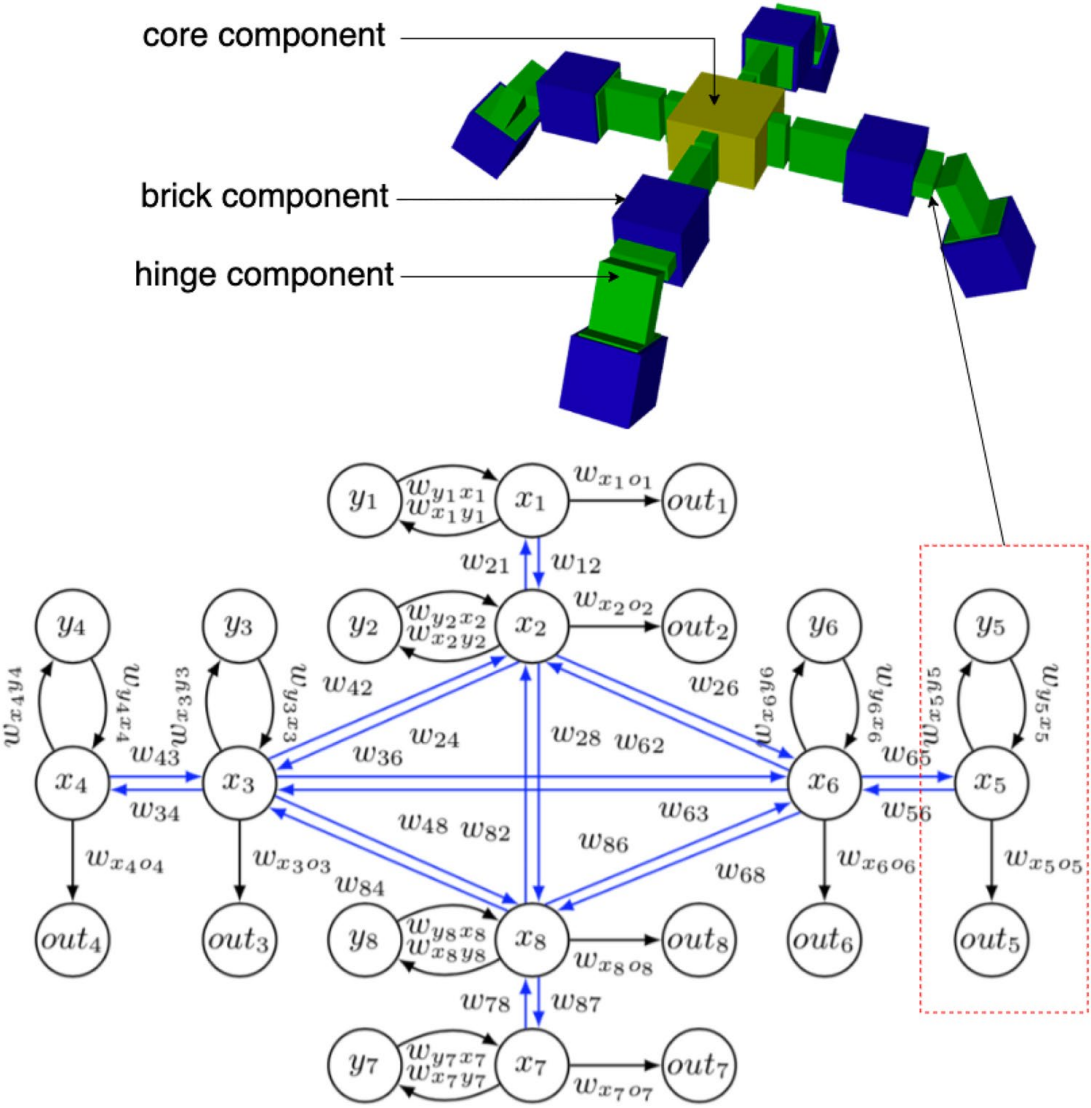
An example of a “+” shape robot and its brain phenotype (CPG network). In our design, the topology of the brain is determined by the topology of the body. The red rectangle is a single CPG which controls a corresponding hinge.

To enable more complex output patterns, connections between CPGs of neighbouring joints are implemented. An example of the CPG network of a “+” shape robot is shown in Fig. 11. Two joints are said to be neighbours if their distance in the morphology tree is less than or equal to two. Consider the $i_{th}$ joint, and $\mathscr {N}_i$ the set of indices of the joints neighbouring it, $w_{ij}$ the weight of the connection between $x_i$ and $x_j$. Again, $w_{ij}$ is set to be $-w_{ji}$. The extended system of differential equations becomes Eq. (1).

1 $$\begin{aligned} \begin{aligned} \dot{x}_i&= w_i y_i + \sum _{j \in \mathscr {N}_i} w_{ji} x_j, \hspace{1cm} \dot{y}_i&= -w_i x_i \end{aligned} \end{aligned}$$

2 $$\begin{aligned}{} & {} out_{(i,t)}(x_{(i,t)}) = \frac{2}{1+e^{-2x_{(i,t)}}} - 1 \end{aligned}$$

Because of this addition, *x* neurons are no longer bounded between $[-1,1]$. For this reason, we use the hyperbolic tangent function (*tanh*) as the activation function of $out_i$-neurons (Eq. 2).

#### Brain genotype

In biological organisms, including humans, not all genes are actively expressed or used at all times. Gene expression regulation allows cells to control which genes are turned on (expressed) or off (silenced) in response to various internal and external factors. Inspired by this, we utilize a fixed size array-based structure for the brain’s genotypic representation to map the CPG weights. It is important to notice that not all the elements of the genotype matrix are going to be used by each robot. This means that their brain’s genotype can carry additional information that could be exploited by their children with different morphologies.

The mapping is achieved via direct encoding, a method chosen specifically for its potential to enable reversible encoding in future stages. Every modular robot can be represented as a 3D grid in which the core module occupies the central position and each module’s position is given by a triple of coordinates. When building the controller from our genotype, we use the coordinates of the joints in the grid to locate the corresponding CPG weight. To reduce the size of our genotype, instead of the 3D grid, we use a simplified 3D in which the third dimension is removed. For this reason, some joints might end up with the same coordinates and will be dealt with accordingly.

Since our robots have a maximum of 10 modules, every robot configuration can be represented in a grid of $21 \times 21$. Each joint in a robot can occupy any position of the grid except the center. For this reason, the possible positions of a joint in our morphologies are exactly $(21 \cdot 21) - 1=440$. We can represent all the internal weights of every possible CPG in our morphologies as a 440-long array. When building the phenotype from this array, we can simply retrieve the corresponding weight starting from a joint’s coordinates in the body grid.

To represent the external connections between CPGs, we need to consider all the possible neighbours a joint can have. In the 2-dimensional grid, the number of cells in a distance-2 neighbourhood for each position is represented by the Delannoy number $D(2,2) = 13$, including the central element. Each one of the neighbours can be identified using the relative position from the joint taken into consideration. Since our robots can assume a 3D position, we need to consider an additional connection for modules with the same 2D coordinates.

**Figure 12:**
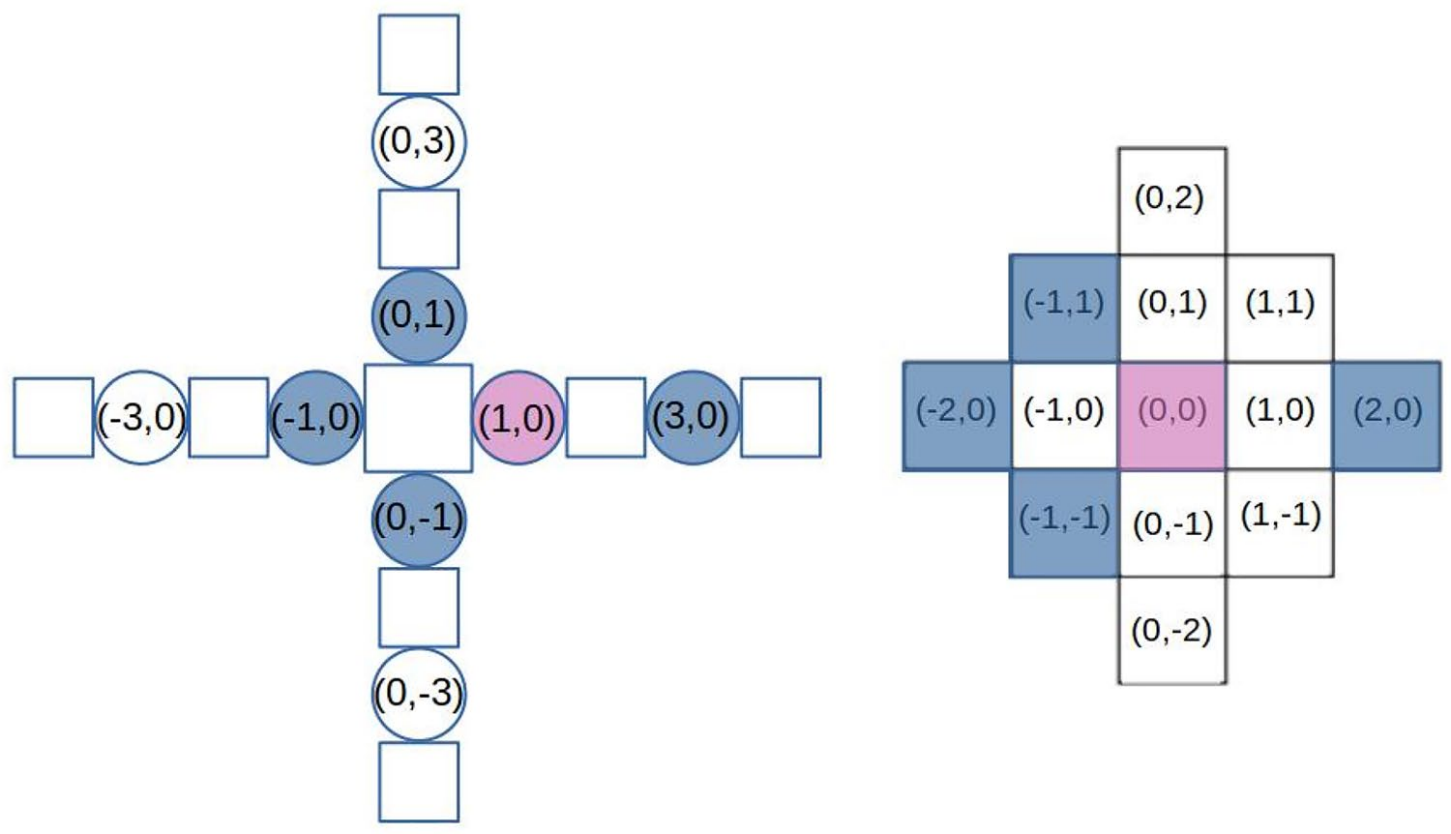
Brain genotype to phenotype mapping of a “+” shape robot. The left image (brain phenotype) shows the schema of the “+” shape robot with the coordinates of its joints in the 2D body grid. The right image (brain genotype) is the distance 2 neighbour of the joint at (1,0). The coordinates reported in the neighbourhood are relative to this joint. The CPG weight of the joint is highlighted in purple and its 2-distance neighbors are in blue.

To conclude, for each of the 440 possible joints in the body grid, we need to store 1 internal weight for its CPG, 12 weights for external connections, and 1 weight for connections with CPGs at the same coordinate for a total of 14 weights. The genotype used to represent the robots’ brains is an array of size $440 \times 14$. An example of the brain genotype of a “+” shape robot is shown in Fig. 12.

The recombination operator for the brain genotype is implemented as a uniform crossover where each gene is chosen from either parent with equal probability. The new genotype is generated by essentially flipping a coin for each element of the parents’ genotype to decide whether or not it will be included in the offspring’s genotype. In the uniform crossover operator, each gene is treated separately. The mutation operator applies a Gaussian mutation to each element of the genotype by adding a value, with a probability of 0.8, sampled from a Gaussian distribution with 0 mean and 0.5 standard deviation.

### Integrated evolution and learning

**Figure 13:**
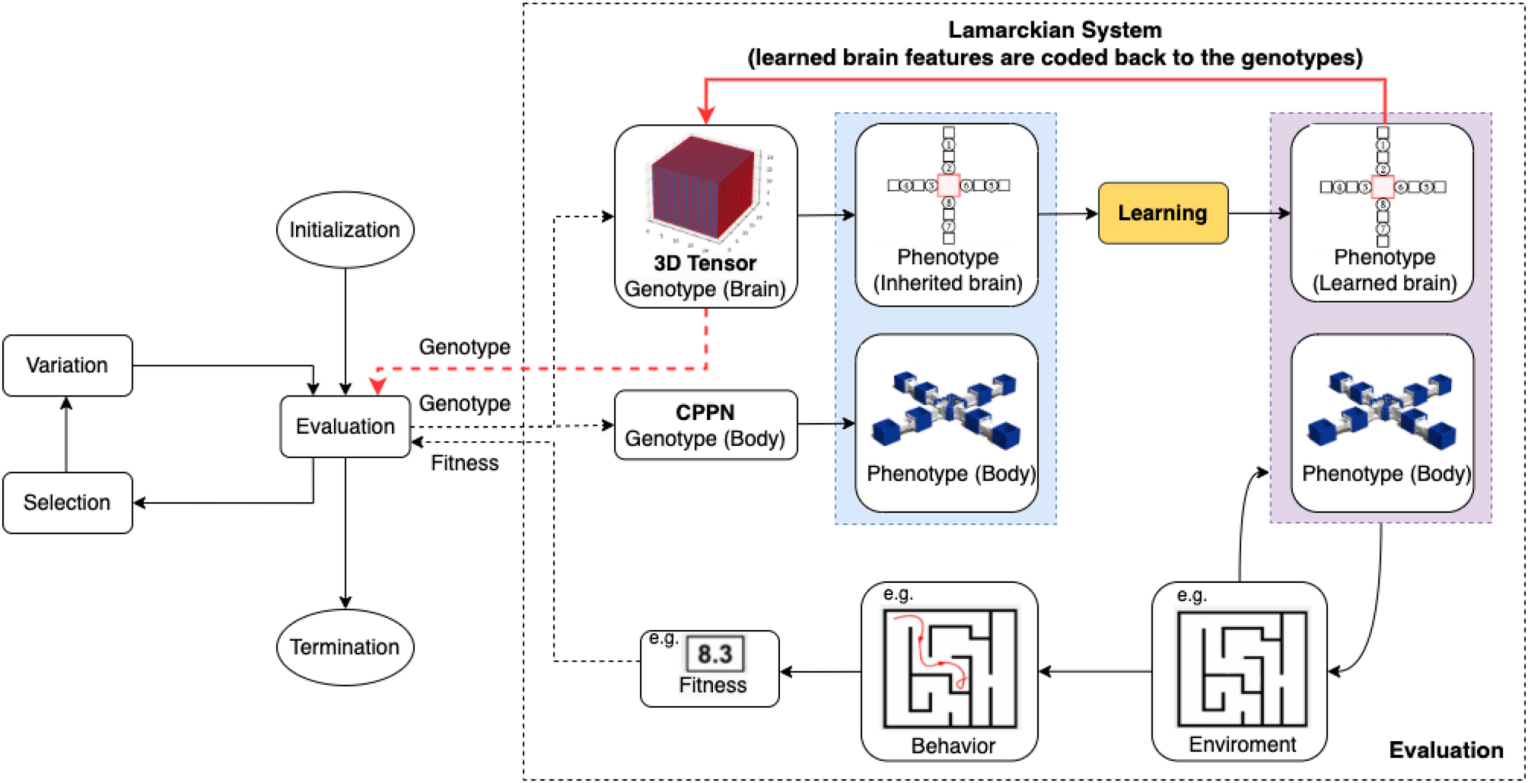
Generic framework for optimizing robots via two interacting adaptive processes, evolution and learning. The evolutionary loop (left) optimizes robot morphologies and controllers simultaneously using genotypes that encode both morphologies and controllers. The learning process (yellow box) optimizes the controller for a given morphology. In the case of a Lamarckian system, the learned traits of the brain are coded back to the genotype, thus making them inheritable.

The complete integrated process of evolution and learning is illustrated in Fig. 13, while Algorithm 1 displays the pseudocode. As explained briefly in the Results section, immediately after birth (steps 7 and 8 in Algorithm 1), all ‘newborn’ robots undergo a learning process that aims to optimize the inherited weights of their controllers (steps 9 to 13 in Algorithm 1) and their fitness is evaluated after learning (step 14 in Algorithm 1). The dotted box on the right hand side of Fig. 13 shows this visually. The newly produced body genotype and brain genotype are used to create the new body phenotype and new brain phenotype that together form a new robot (purple box). The learning algorithm (yellow block) searches through the space of possible weight vectors with the learned brain as a result. The combination of the inherited body and the learned brain (blue box) is the ‘learned robot’ that undergoes fitness evaluation. After learning, the fitness achieved with the learned brain is returned to the evolutionary process as the result of the Evaluation module in Fig. 13.

In general, there are no restrictions regarding the learning method; it can be any search algorithm that can search through the space of all brain configurations in an attempt to increase the robots’ performance. Note that for the sake of generality, we distinguish two types of quality testing depending on the context, evolution or learning. Within the evolutionary cycle, a test is called an evaluation (line 2 and line 14) and it delivers a fitness value. Inside the learning cycle, a test is called an assessment (line 11) and it delivers a reward value. This distinction reflects that in general the notion of fitness can be different from the task performance, perhaps more complex involving more tasks, other behavioral traits not related to any task, or even morphological properties.

**Algorithm 1:**
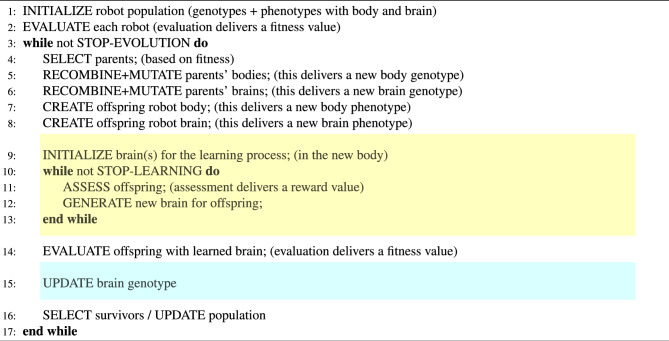
Evolution+Learning.

#### Evolution process

Each generation in the evolutionary process consists of $\mu$ individuals. Given the *n*-th generation $G_n$, the next generation $G_{n+1}$ is created as follows. First, a set of $\lambda$ children is produced by selecting parents using standard binary tournaments with replacement and applying recombination and mutation to the parents. Then the best $\mu -\lambda$ individuals from $G_n$ are selected deterministically as survivors and $G_{n+1}$ is the union of the children and the survivors.

Reproduction is split into two parts, reproduction of the body and reproduction of the brain. The body of a child is created by sexual reproduction: the genotypes of the two parents are recombined and mutated to produce the offspring genotype. The brain of a child is created by asexual reproduction: the brain genotype of the fittest parent undergoes random mutation and the resulting genotype is inherited by the offspring. This choice is based on preliminary experiments that indicated that asexual brain reproduction is the better method, as it resulted in robots with higher fitness.

#### Learning process

The search algorithm we have chosen as a learning method is Reversible Differential Evolution (RevDE), because a recent study on modular robots^73^ demonstrated that RevDE^74,75^ performs and generalizes well as a learner across various morphologies. This algorithm works as follows:

1. Initialize a population with *K* samples (*n*-dimensional vectors), $\mathscr {P}$.
2. Evaluate all *K* samples.
3. Apply the reversible differential mutation operator and the uniform crossover operator. *The reversible differential mutation operator*: Three new candidates are generated by randomly picking a triplet from the population, $(\textbf{w}_i,\textbf{w}_j,\textbf{w}_k)\in \mathscr {P}$, then all three individuals are perturbed by adding a scaled difference in the following manner:
  3 $$\begin{aligned} \begin{aligned} \textbf{v}_1&= \textbf{w}_i + F \cdot (\textbf{w}_j-\textbf{w}_k) \\ \textbf{v}_2&= \textbf{w}_j + F \cdot (\textbf{w}_k-\textbf{v}_1) \\ \textbf{v}_3&= \textbf{w}_k + F\cdot (\textbf{v}_1-\textbf{v}_2) \end{aligned} \end{aligned}$$
  where $F\in R_+$ is the scaling factor. New candidates $y_1$ and $y_2$ are used to calculate perturbations using points outside the population. This approach does not follow the typical construction of an EA where only evaluated candidates are mutated. *The uniform crossover operator*: Following the original DE method^76^, we first sample a binary mask $\textbf{m} \in \{0, 1\}^D$ according to the Bernoulli distribution with probability *CR* shared across *D* dimensions, and calculate the final candidate according to the following formula:
  4 $$\begin{aligned} \textbf{u} = \textbf{m} \odot \textbf{w}_n+(1-m) \odot \textbf{w}_n \end{aligned}$$
  Following general recommendations in literature^77^ to obtain stable exploration behaviour, the crossover probability CR is fixed to a value of 0.9 and according to the analysis provided in^74^, the scaling factor *F* is fixed to a value of 0.5.
4. Perform a selection over the population based on the fitness value and select *K* samples.
5. Repeat from step (2) until the maximum number of iterations is reached.

As explained above, we apply RevDE here as a learning method for ‘newborn’ robots. In particular, it will be used to optimize the weights of the CPGs of our modular robots for the tasks during the Infancy stage. The initial population of $X = 10$ weight vectors for RevDE is created by using the inherited brain of the given robot. Specifically, the values of the inherited weight vector are altered by adding Gaussian noise to create mutant vectors and the initial population consists of nine such mutants and the vector with the inherited weights.

### Task and fitness function

Point navigation requires feedback (coordinates)from the environment passing to the controller to steer the robot. The coordinates are used to obtain the angle between the current position and the target. If the target is on the right, the right joints are slowed down and vice versa.

A robot is spawned at the centre of a flat arena (10 $\times$ 10 m2) to reach a sequence of target points $P_1,..., P_N$. In each evaluation, the robot has to reach as many targets in order as possible. Success in this task requires the ability to move fast to reach one target and then quickly change direction to another target in a short duration. A target point is considered to be reached if the robot gets within 0.01 meters from it. To keep runtimes within practically acceptable limits, we set the simulation time per evaluation to be 40 seconds which allows robots to reach at least 2 targets $P_1(1,-1), P_2(0,-2)$.

The data collected from the simulator is the following:

- The coordinates of the core component of the robot at the start of the simulation are approximate to $P_0 (0,0)$;
- The coordinates of the robot at the end of the simulation $P_T(x_T,y_T)$;
- The coordinates of the target points $P_1(x_1,y_1)$... $P_n(x_n,y_n)$.
- The coordinates of the robot, sampled during the simulation at 5Hz, allow us to plot and approximate the length of the path *L*.

The fitness function is designed to maximize the number of targets reached and to minimize the path length.

5 $$\begin{aligned} F=\sum _{i=1}^{k}dist(P_i,P_{i-1}) +(dist(P_k,P_{k-1}) - dist(P_T,P_k)) - \omega \cdot L \end{aligned}$$

where *k* is the number of target points reached by the robot at the end of the evaluation, and *L* is the path travelled. The first term of the function is a sum of the distances between the target points the robot has reached. The second term is necessary when the robot has not reached all the targets and it calculates the distance travelled toward the next unreached target. The last term is used to penalize longer paths and $\omega$ is a constant scalar that is set to 0.1 in the experiments. E.g., a robot just reached 2 targets, the maximum fitness value will be $dist(P_1,P_0)+(dist(P_2,P_1)-dist(P2,P2))-0.1\cdot L=\sqrt{2}+\sqrt{2}-0.2\cdot \sqrt{2} \approx 2.54$ (*L* is shortest path length to go through $P_1$ and $P_2$ which is equal to $2\cdot \sqrt{2}$).

### Experimental setup

We use a Mujoco simulator-based wrapper called Revolve2 (https://github.com/ci-group/revolve2) to run experiments. For the evolutionary process we set $\mu =50$ and $\lambda = 25$. The evolutionary process is terminated after 30 generations, resulting in $25+25\cdot 30 = 775$ fitness evaluations in total. To calculate the robots’ fitness we use a test procedure simulating a robot for 40 simulated seconds for the point navigation task.

Using RevDE for learning good weights for the robot brains adds 280 extra fitness evaluations for each robot. (We equate rewards for learning (step 11 in Algorithm 1) and fitness for evolution (step 14 in Algorithm 1).) This number is based on RevDE starting with $K = 10$ initial weight vectors as samples and running for 10 iterations. The first iteration contains 10 weight vectors, and from the second iteration onwards each iteration creates 30 new vectors, resulting in a total of $10 + 30 \cdot (10-1)= 280$ evaluations.

To sum up, for running the experiments we perform $775\cdot 280\cdot 2= 434,000$ fitness evaluations which amount to $434,000 \cdot 40/60/60=4,822$ hours of simulated time. To get a robust assessment of the performance all experiments are repeated 20 times independently. In practice, it takes about 4.5 days to run two instances in parallel on two 64-core processor workstations. The experimental parameters we used in the experiments are described in Table 1.

**Table 1:** Main experiment parameters.

Evolution parameters	Value	Description
Population size $\mu$	50	Number of individuals per generation
Offspring size $\lambda$	25	Number of offspring produced per generation
Tournament size	2	Number of individuals used in the parent selection
Mutation rate	0.8	Probability of mutation for individuals
Crossover rate	0.8	Probability of crossover for individual’s body
Max. no. of generations	30	Termination condition for the evolutionary runs
Evaluation time	40	Duration of evaluation in seconds
Repetitions	20	Number of repetitions per experiment

Learning parameters	Value	Description
Population size *K*	10	Population size in RevDE
Scaling factor *F*	0.5	Scaling factor for RevDE
Crossover rate *CR*	0.9	Probability of crossover in RevDE
Max. no. of iterations	10	Termination condition for RevDE
Evaluation time	40	Duration of evaluation in seconds

## Data Availability

The code for replicating this work and carrying out the experiments is available online: https://shorturl.at/epzGS. A short video providing a visual overview of our research is available at https://shorturl.at/ahETW.
